# Short-term high-fat and high-carb diet effects on glucose metabolism and hedonic regulation in young healthy men

**DOI:** 10.3389/fnut.2024.1469230

**Published:** 2024-10-29

**Authors:** Marcel Pointke, Frank Strenge, Dawid Piotrowski, Anika Matteikat, Svenja Meyhöfer, Sebastian M. Meyhöfer, Rodrigo Chamorro, Britta Wilms

**Affiliations:** ^1^Medical Clinic I, University of Lübeck, Lübeck, Germany; ^2^Center of Brain, Behavior and Metabolism, University of Lübeck, Lübeck, Germany; ^3^German Center for Diabetes Research (DZD), Munich, Germany; ^4^Novo Nordisk Pharma GmbH, Mainz, Germany; ^5^Department of Nutrition, Faculty of Medicine, University of Chile, Santiago, Chile

**Keywords:** macronutrient, diet, glucose homeostasis, appetite, hedonic hunger

## Abstract

**Background:**

Daily dietary intake of macronutrients and energy is closely associated with long-term metabolic health outcomes, but whether 24-h nutritional intervention under isocaloric conditions leads to changes in metabolism remains unclear. Moreover, the short-term effect of diets with different macronutrient composition on hedonic appetite regulation is less clear.

**Methods:**

This study examined the impact of an acute high-fat (F+) and high-carbohydrate (C+) diet on glucose metabolism and hedonic regulation of food intake in young healthy men under controlled conditions. Using a cross-over design, 19 male participants received a one-day isocaloric diet with different macronutrient composition (F+ = 11% carbohydrates, 74% fat; C+ = 79% carbohydrates, 6% fat) compared to a control diet (CON = 55% carbohydrates, 30% fat). Protein content was set at 15% of energy in all diets. The feeling of hunger, as well as “liking” and “wanting” for foods, was assessed through visual analog scales, and blood samples for glucose, insulin, and cortisol levels were assessed repeatedly during the experimental day. An intravenous glucose tolerance test was conducted the next morning.

**Results:**

Postprandial glucose and insulin levels were lowest in F+ over the 24 h. Except for dinner, the CON diet showed the highest mean values in glucose. F+ diet improved insulin resistance, lowering Homeostatis Model Assessment Insulin Resistance (HOMA-IR) values. Changes in hedonic regulation of food intake were not observed during the intervention between the diets, except for higher feelings of satiety under the CON diet.

**Conclusion:**

An acute, isocaloric, high-fat diet improved insulin resistance even in healthy individuals but did not affect hedonic food intake regulation. Macronutrient composition modulate glucose metabolism even under short-term (24-h) and isocaloric diets, which should be considered for personalized preventive dietary treatments.

## 1 Introduction

In the Western world, nutrition is not only a fundamental human need but has also developed into a lifestyle issue. Food and nutrition have become a trend, a component of identity, and a status symbol for many people. Various nutritional advice and diets are supposed to improve health outcomes and quality of life. For young adults, social media, in particular, is of immense importance in influencing food choices (1, 2). However, valid data to assess the effect of these different diet strategies on metabolic parameters (3) and food intake regulation (4) are often lacking.

Although many dietary approaches are popular, such as Dietary Approaches to Stop Hypertension, low-fat, moderate-carbohydrate, low-carbohydrate, high-protein, Mediterranean, Paleolithic, vegetarian, low-glycemic index/load, low-sodium, New Nordic Diet, their overall benefit as well as their risks have not yet been conclusively established (5). Often, the macronutrient composition is far from current recommendations, i.e., carbohydrates of at least 50% of energy intake (6), fats of 30% of energy intake (7), and proteins in adults <65 years: 0.8 g/kg body weight per day (8).

It has been a significant focus in nutritional research, especially concerning diet-related noncommunicable diseases (NCDs), such as obesity and type 2 diabetes (T2D). The extent of restriction or increased proportion of one macronutrient in the diet impacts metabolic health. The acute effects of specific diets and type of meals with dissimilar carbohydrate and fat composition on metabolic response has been shown in both patients and healthy humans (9, 10). The metabolic response to those diets is mixed, with factors such as ethnicity and race being relevant for observed outcomes (11–13) Diurnal variations in between meals (i.e., differential metabolic results to meals with different carbohydrate/fat composition across the day) have also been shown (10, 14). Other approaches such as a high-fat, ketogenic, low-carbohydrate diet has been also investigated in animal and human models (15, 16).

Evidence suggests that the long-term consequences of chronic hypercaloric, i.e., energy intake exceeding energy expenditure, high-fat diet can lead to weight gain, insulin resistance, and the manifestation of T2D (17). The macronutrient composition of the diet can also influence metabolism (18). For instance, sugar-sweetened beverages are a major source of added sugar and have been shown to accelerate weight gain and develop T2D and cardiovascular diseases (19, 20). Results from epidemiologic studies comparing the effects of low-carbohydrate and low-fat diets on metabolic risk factors and weight loss in overweight and obese adults are, at least in part, contradictory. The meta-analysis by Lei et al. (21) included overweight and obese participants with or without basic diseases and showed that both diets positively affected weight loss and improved metabolic risk factors, lasting up to 2 years to the same extent. Previous studies, even in healthy participants, have shown that low-carbohydrate diets are more effective for weight loss than low-fat diets (22, 23).

Hedonic factors in general influence subjective well-being, which can be influenced by the consumption of certain foods or diets, also known as comfort food. Hedonic value is highly influenced by the macronutrient composition in the diet and high-fat and high-carbohydrate foods are strongly associated with pleasure and satisfaction (24, 25). The propensity to consume is increased by the activation of reward centers in the brain, which in turn impacts eating habits and health (26). Studies show that diets with a balanced macronutrient distribution are perceived as tasty and filling. This promotes their long-term acceptance and adherence (27, 28). This contrasts with more extreme diets, which may be popular for a short time but are often not sustained due to their lack of hedonic value (29).

The impact of a short-term isocaloric diet, either high-carbohydrate (C+) or high-fat (F+), on metabolic and hedonic food intake regulation remains poorly understood. Therefore, we aimed to evaluate the effects of a C+ and F+ diet compared to a control (CON) diet not only on metabolism, i.e., glucose metabolism but also on hedonic food regulation over 24-h under standardized diets in young, healthy individuals.

## 2 Materials and methods

### 2.1 Participants

Twenty-two healthy, normal-weight men aged 18 to 40 years (mean ± SEM, 25.1 ± 0.8 years) with a body-mass index (BMI) between 18.5 to 24.9 kg/m^2^ (22.6 ± 0.6 kg/m^2^) were enrolled in this study. Participants underwent a screening visit, including a physical examination, electrocardiogram, health status questionnaires, and dietary and sleep habits assessment. A venous blood sampling was performed to determine blood count, liver and kidney status, lipid and glucose metabolism parameters, and coagulation diagnosis. Participants were excluded if they had hyper- as well as hypothyroid metabolic status, dyslipidemia, coagulation disorders, elevated fasting glucose levels, and a positive family history of diabetes mellitus. Further exclusion criteria were regular medication use, disturbed sleep–wake rhythm (assessed by a one-night polysomnographic evaluation), working night shifts, active competitive sports, and having special dietary habits. Participants were not allowed to participate in any other study or donate blood during study participation.

At each experimental day, physical activity, sleep pattern, diet, and subjective feelings were assessed by a structured interview the day before to ensure that participants adhered to the above criteria. Body composition was evaluated by Air Displacement Plethysmography (BOD POD, COSMED, Germany).

#### 2.1.1 Sample size

Sample size calculation was performed based on our previously collected experimental data. Given that the general study design of the current study was comparable to our previous studies (30, 31), namely a repeated measures crossover design conducted under well-standardized laboratory conditions and with homogeneous study groups, we assumed a high effect size for the impact of isocaloric diets with an extreme macronutrient composition on metabolic and behavioral parameters in healthy men under these conditions. *A priori* power analysis using G*Power for ANOVA with repeated measures and within-between interaction revealed that a sample size of 18 subjects was required to achieve a high effect size (f2 = 0.4), alpha = 0.05, beta = 0.8, correlation among repeated measures = 0.6, and non-sphericity correction = 1.

The ethics committee of the University of Lübeck approved the study protocol (EC 16-343; approved April 06, 2017) according to the Declaration of Helsinki. Each participant provided oral and written informed consent.

### 2.2 Study design and experimental protocol

**Figure 1:**
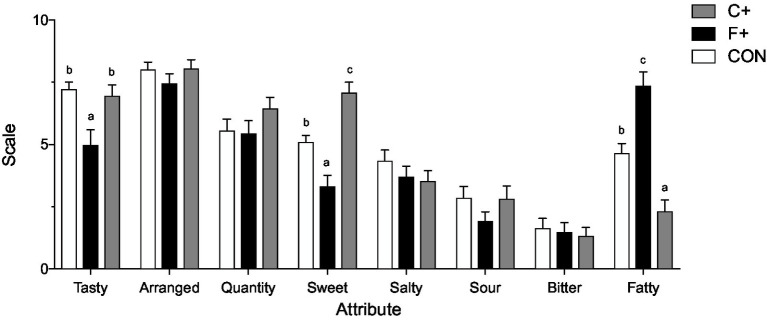
Data as mean ± SEM. Sensory evaluation of control diet (CON), high-fat diet (F+), and high-carbohydrate diet (C+). ANOVA with Tukey’s HSD test, significant differences (*p* < 0.05).

This was a randomized, balanced cross-over study in which participants underwent three experimental diets over two and a half days each, spaced 1–3 weeks apart (see Figure 1): (i) C+ diet, with 79% carbohydrates and 6% fat, (ii) F+ diet, with 11% carbohydrates and 74% fat, and (iii) CON diet, with 55% carbohydrates and 30% fat. The protein content was set at 15% of energy in all diets. Energy density of CON and C+ was 5.6 kcal/g and 5.4 kcal/g, respectively, while F+ was characterized by an energy density of 9.3 kcal/g. In CON, the macronutrient composition corresponded to the evidence-based guidelines of the German Nutrition Society (6–8).

The energy content of each diet was based on calculated individual total energy expenditure (TEE) upon predicted resting energy expenditure (REE) by the Harris-Benedict equation (32) and a physical activity level of 1.3, reflecting low intense activities under the in-lab context (33). Participants were not allowed to leave the research unit during the experimental days and were instructed not to exert themselves physically. They were allowed to read, play games, watch movies, and listen to music. Participants were served water *ad libitum* and tea or coffee without caffeine during the meals. Snacks were not allowed.

On each experimental visit, participants arrived at the research unit at 07:00 p.m. (day 1, Figure 1). An intravenous catheter was inserted into a vein of the participant’s non-dominant distal forearm to allow the drawing of blood samples. Regardless of the condition, an isocaloric standardized dinner with a macronutrient composition of the CON diet was served at 08:00 pm the evening before, and they were instructed to consume meals in their entirety. Participants went to bed, and light was turned off at 11:00 p.m. (nights 1 and 2). Sleep was recorded by polysomnography (Nihon Kohden, Rosbach, Germany) each night. Recordings were scored offline in 30 s-epochs by two independent researchers using standard criteria (34). In the morning of day 2 (07:00 a.m.), participants were woken up and received a standardized meal at 08:30 a.m., 1:30 p.m., and 08:00 p.m., the macronutrient ratio adapted to the respective C+, F+ or CON diet.

Respective questionnaires and tests on “liking” and “wanting” were carried out before and after each meal, and blood samples were collected. REE was measured on the morning of day 2 and day 3 directly after participants’ awakening by indirect calorimetry using a ventilated hood system (Vmax 29n, SensorMedics, Höchberg, Germany) for 30 min. Before measurement, gas analyzers were calibrated with standard gas concentration (16% O_2_ + 4% CO_2_, 26% O_2_). During measurement, participants were lying in a supine position awake, quiet, and motionless. The respiratory exchange ratio was calculated as ratio of V’CO_2_ and V’O_2_.

Right after REE assessment on day 3, a second IV catheter was inserted into the participant’s contralateral distal forearm vein. Glucose homeostasis was evaluated by an intravenous glucose tolerance test (ivGTT). A glucose bolus of 1.5 mL/kg body weight of a 20% glucose solution was injected. Blood samples were collected at −5, 0, 2, 4, 6, 8, 10, 15, 30, 45, and 60 min after glucose loading (30).

### 2.3 Laboratory chemical determinations

Blood glucose level was determined immediately by EKF Diagnostics’ Biosen C-Line (EKF Diagnostics GmbH, Barleben, Germany). According to the manufacturer, the within-assay variation was below 1.5% (mean 12 mmol/L). Insulin and cortisol levels were determined in serum using the IMMULITE 2000 XPi immunoassay system (SIEMENS Healthineers, Germany) with detection limits and average within-assay coefficients of variation of 2.0 mUI/mL and 5.3% (insulin) and 5.5 nmol/L and 7.4% (cortisol). All values were determined from stored (−80°C) serum samples. The homeostasis model assessment index of insulin resistance (HOMA-IR) was calculated from fasting glucose and insulin values (35). The insulinogenic index is determined as a marker for beta-cell function from the rise in glucose and insulin levels during the first 30 min of the ivGTT.

### 2.4 Assessment of hedonic regulation of food intake, processing of eating stimuli, eating behavior, and sensory perception

Subjective feelings of hunger and satiety were determined before and after meals (36) using a 100 mm visual analog scale (VAS) anchored from “not at all” to “maximum.”

After that, participants rated a series of food stimuli using a test of explicit “liking” and “wanting” for foods (23). In brief, 42 food images differing in energy content (i.e., high- or low-energy) and taste (i.e., non-sweet or sweet) were presented using a personal computer using the Matlab platform (Matlab, v7.5.0, The MathWorks, Inc., Natick, MA, United States). The food items were classified according to their energy content and separated into non-sweets (i.e., high-energy, fat-rich foods) and sweets (i.e., high-energy, sugar-rich foods). The participants were asked, “How pleasant would you find the taste of this food?” to assess the liking aspect, and “How much do you want some of this food right now?” for the wanting aspect, respectively. All items were rated on a Likert scale from 1 (“not at all”) to 5 (“very much”).

Participants completed the German version (FEV = Fragebogen zum Essverhalten) of the three-factor eating questionnaire (TFEQ) (37, 38) at all conditions on the morning of day 2. The first scale captures the extent of cognitive control of eating behavior in terms of restrained eating aimed at limiting food intake. The second scale measures the extent to which eating behavior can be disturbed by situational stimuli or the person’s emotional state. The third scale captures vulnerability to orthorexia nervosa, a pathological preoccupation with healthy eating.

Participants were asked to rate their meals sensory from the previous day on day 3. In addition to four basic tastes (sweet, sour, salty, bitter), the attribute “fatty” was rated. Furthermore, participants should “quantity” the presented amount, the arrangement of the menu, and “tasty” the overall impression. Ratings were performed on a 100 mm VAS anchored “dislike extremely” (0) and “like extremely” (10).

### 2.5 Statistical evaluation

Nineteen men completed all three experimental diets, and data analysis was performed on these data sets. Analyses were performed using SPSS software, version 29.0 for Mac (IBM Inc., Chicago, IL), and Prism software, version 7.0e (GraphPad Software Inc., La Jolla, CA). Data were analyzed using analysis of variance (ANOVA) with repeated measures according to the general linear model and the factors “condition” (CON vs. F+ vs. C+) and “time” (time course of measurement results). For single comparisons between two measurement points, the paired *t*-test is used. In all analyses, a *p*-value < 0.05 was considered significant.

## 3 Results

### 3.1 Characteristics of the study population

Participants showed no significant changes in body mass, BMI, and body fat during the study (all *p* ≥ 0.05; Table 1). REE was not different between day 2 and day 3, independent of the condition (all p ≥ 0.05). At day 3, RQ was not different between CON and F+ (*p* = 0.15), but significant changes to C+ diet RQ was 0.83 ± 0.01 (*p* ≤ 0.001). In the evaluation of the TFEQ, no differences were found between all three diets (Table 1). The polysomnography results of the two nights showed that the sleep stages did not differ between the three diets.

**Table 1:** Measures of body composition, indirect calorimetry, and aspects of eating behavior.

	CON	F+	C+	*p* value
Age (years)	25.1 ± 0.8			–
Body mass (kg)	75.5 ± 2.1	75.2 ± 2.2	75.6 ± 2.0	0.997
BMI (kg/m^2^)	22.6 ± 0.4	22.5 ± 0.4	22.6 ± 0.4	0.973
Body fat (%)	17.7 ± 1.0	17.7 ± 1.2	18.1 ± 1.1	0.984
REE (kcal)^Day2^	1,550 ± 49	1,528 ± 36	1,541 ± 44	0.941
REE (kcal)^Day3^	1,489 ± 39	1,599 ± 43	1,537 ± 48	0.210
RQ^Day2^	0.82 ± 0.01	0.82 ± 0.01	0.81 ± 0.01	0.688
RQ^Day3^	0.78 ± 0.01^a^	0.76 ± 0.01^a^	0.83 ± 0.01^b^	<0.001
TFEQ Scale 1	5.8 ± 0.6	6.0 ± 0.6	6.0 ± 0.7	0.963
TFEQ Scale 2	4.0 ± 0.4	4.4 ± 0.3	4.0 ± 0.4	0.631
TFEQ Scale 3	2.3 ± 0.4	2.3 ± 0.3	2.4 ± 0.3	0.951

Data are mean ± SEM. CON = Control diet, F+ = High-fat diet, C+ = High-carbohydrate diet, REE = resting energy expenditure, RQ = Respiratory quotient, Three-factor eating questionnaire (TFEQ), Scale 1 = Cognitive restraint scale, Scale 2 = Disinhibition scale, Scale 3 = Orthorexia scale; values are mean ± SD, *p* values from repeated-measures ANOVA with Tukey’s HSD test. The superscript letters “a, b” indicate the significant differences that could be determined between the groups.

### 3.2 Diet intervention

Diets’ total energy intake was closely matched (Table 2), and total protein intake (15% energy) was constant across diets. Carbohydrates, fiber, and sugar proportion were significantly higher in C+ compared with F+ and CON, respectively (Table 2). The proportion of total fat and fatty acids was significantly lowest in C+, except for polyunsaturated fatty acids, which were highest in F+. Energy density was highest in F+ with no differences between C+ and CON (*p* < 0.001).

**Table 2:** Nutrient intake of tested diets.

	CON	F+	C+
Energy (kcal)	2,154 ± 61	2,125 ± 59	2,136 ± 60
Protein (g)	72.83 ± 2.1	79.67 ± 2.2	75.09 ± 2.1
Carbohydrate (g)	309.2 ± 8.7^b^	79.80 ± 2.2^a^	432.9 ± 12.1^c^
Fiber (g)	15.03 ± 0.4^a^	19.36 ± 0.5^b^	23.47 ± 0.7^c^
Sugar (g)	133.8 ± 7.6^b^	31.21 ± 2.0^a^	178.0 ± 5.0^c^
Fat (g)	74.2 ± 2.1^b^	171.1 ± 4.7^c^	15.6 ± 0.4^a^
Saturated fat (g)	29.9 ± 0.8^b^	77.75 ± 2.2^c^	3.0 ± 0.1^a^
Monounsaturated fat (g)	17.9 ± 0.5^b^	46.5 ± 1.5^c^	0.77 ± 0.0^a^
Polyunsaturated fat (g)	15.7 ± 0.4^c^	9.1 ± 0.3^b^	0.88 ± 0.0^a^
Cholesterol (mg)	166.2 ± 4.7^b^	464.2 ± 13.1^c^	32.9 ± 0.9^a^

Data are mean ± SEM. CON = Control diet, F+ = High-fat diet, C+ = High-carbohydrate diet. Two-way ANOVA with Tukey’s HSD test; significant differences (*p* < 0.05). The superscript letters “a, b,c” indicate the significant differences that could be determined between the groups.

### 3.3 Food choice and sensory evaluation

Regarding food choice data, 52.6% of participants consumed bread daily or more often per day, and rice and pasta were the second most consumed by 57.9% several times per week, thus representing the primary source of carbohydrates (Supplementary Figure A1). Participants preferred high-fat milk, dairy products, and fats over low-fat options. 79% of participants consumed meat one or several times per week, and again, the proportion of high-fat meat products was higher than low-fat options (Supplementary Figure A1). Only 26.3% of participants reported consuming vegetables or legumes daily or more often per day. The proportion of fruits was 31.6% during the same period. Sweets were chosen infrequently by participants in their food choices. Over 60% reported eating cakes, puddings, or ice cream rarely or several times a month (Supplementary Figure A1).

The sensory evaluation of meals from the different diets revealed that C+ was perceived as sweeter 7.1 ± 0.43 (*p* ≤ 0.001), and the F+ diet as fattier 7.4 ± 0.55 (*p* ≤ 0.001) (Figure 1) as compared to the other diets. When rating the attribute for overall liking “tasty,” both CON and C+ were rated higher than F+ (*p* = 0.002).

### 3.4 Hunger—and liking for foods

**Figure 2:**
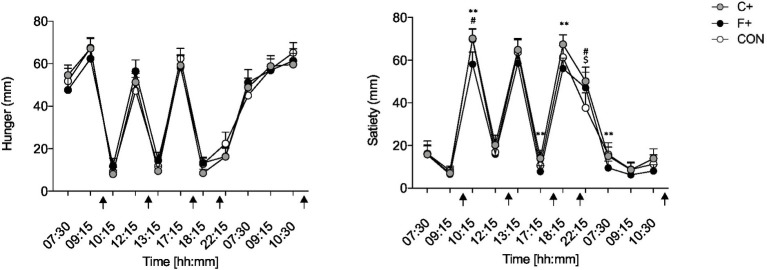
Data as mean ± SEM. Overall hunger (A) and satiety (B) (0–100 mm) of control diet (CON), high-fat diet (F+), and high-carbohydrate diet (C+). Two-way ANOVA for repeated measurements and paired *t*-test for paired samples. *F+ vs. C+, #CON vs. F+, $CON vs. C+. *, #, $ *p* ≤ 0.05; **, ##, $$ *p* ≤ 0.01; ***, ###, $$$ *p* ≤ 0.001. Black arrow: food intake.

Subjective feelings of hunger and satiety between diets are shown in Figure 2. As expected, satiety was highest immediately after food intake, whereas hunger was minimal. As the postprandial period increased, the feeling of hunger increased, while the feeling of satiety decreased (*p* ≤ 0.001 for ANOVA time effect). Between conditions, lower feelings of satiety during the day than C+.

**Figure 3:**
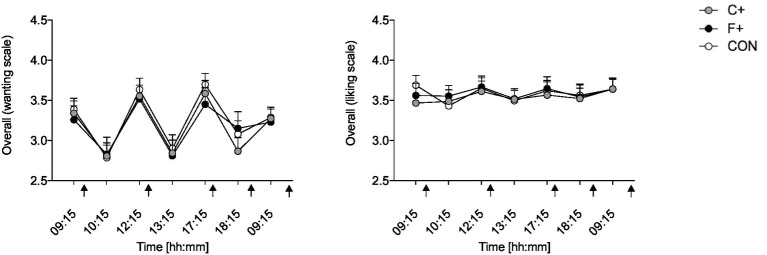
Overall “liking” and “wanting” for foods in the different diet groups (CON, C+, F+); scale from 1 = not at all, 5 = extremely. Data as mean ± SEM; two-way ANOVA for repeated measurements and dependent *t*-test for paired samples. Black arrow: food intake.

“Liking” and “wanting” were not different between diets over the day (both *p* > 0.446 for ANOVA time × condition interaction) (Figure 3). The condition did not impact “liking” or “wanting” for different food categories over the day (all *p* > 0.509 for ANOVA condition × time interaction).

### 3.5 Daily profile of parameters of glucose homeostasis

**Figure 4:**
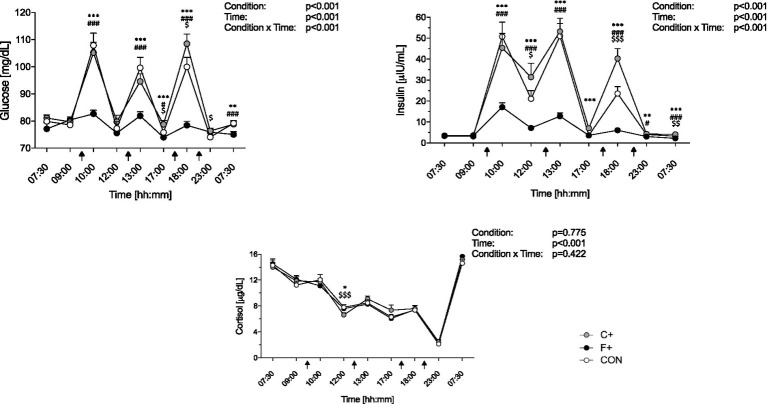
Daily profile of glucose **(A)**, insulin **(B)**, and cortisol **(C)** among the three diets (CON, F+, C+). Data as mean ± SEM. Two-way ANOVA for repeated measures and dependent *t*-test for paired samples. *F+ vs. C+, #CON vs. F+, $CON vs. C+. *, #, $ *p* ≤ 0.05; **, ##, $$ *p* ≤ 0.01; ***, ###, $$$ *p* ≤ 0.001.

Glucose, insulin, and cortisone daily profiles for the three diets are shown in Figure 4. Both glucose and insulin levels increased after meals and decreased postprandially (all *p* < 0.001 for ANOVA time × condition intervention), with the lowest levels found during F+ (*p* < 0.001 for ANOVA condition effect; Figures 4A,B). Pairwise comparison revealed differences in glucose levels between CON and C+ (*p* ≤ 0.05) (Figure 4A). Postprandial glucose levels were significantly higher in CON and C+ than in F+ (*p* ≤ 0.001). The insulin daily profile revealed the lowest levels in F+ as compared to C+ and CON, respectively (*p* < 0.001 for ANOVA time x condition interaction; Figure 4B). Subsequent pairwise comparison of single time points indicates differences for six-time points for CON compared to F+ (all *p* ≤ 0.001), and seven for F+ compared to C+ (all *p* ≤ 0.01), respectively (Figure 4B). In addition, insulin levels differ at three-time points for CON compared to C+ (all *p* < 0.05). Cortisol levels steadily decreased over the time course for all diets (*p* ≤ 0.001 for ANOVA time effect), independent of condition (*p* = 0.442 for ANOVA time x condition interaction; Figure 4C).

**Figure 5:**
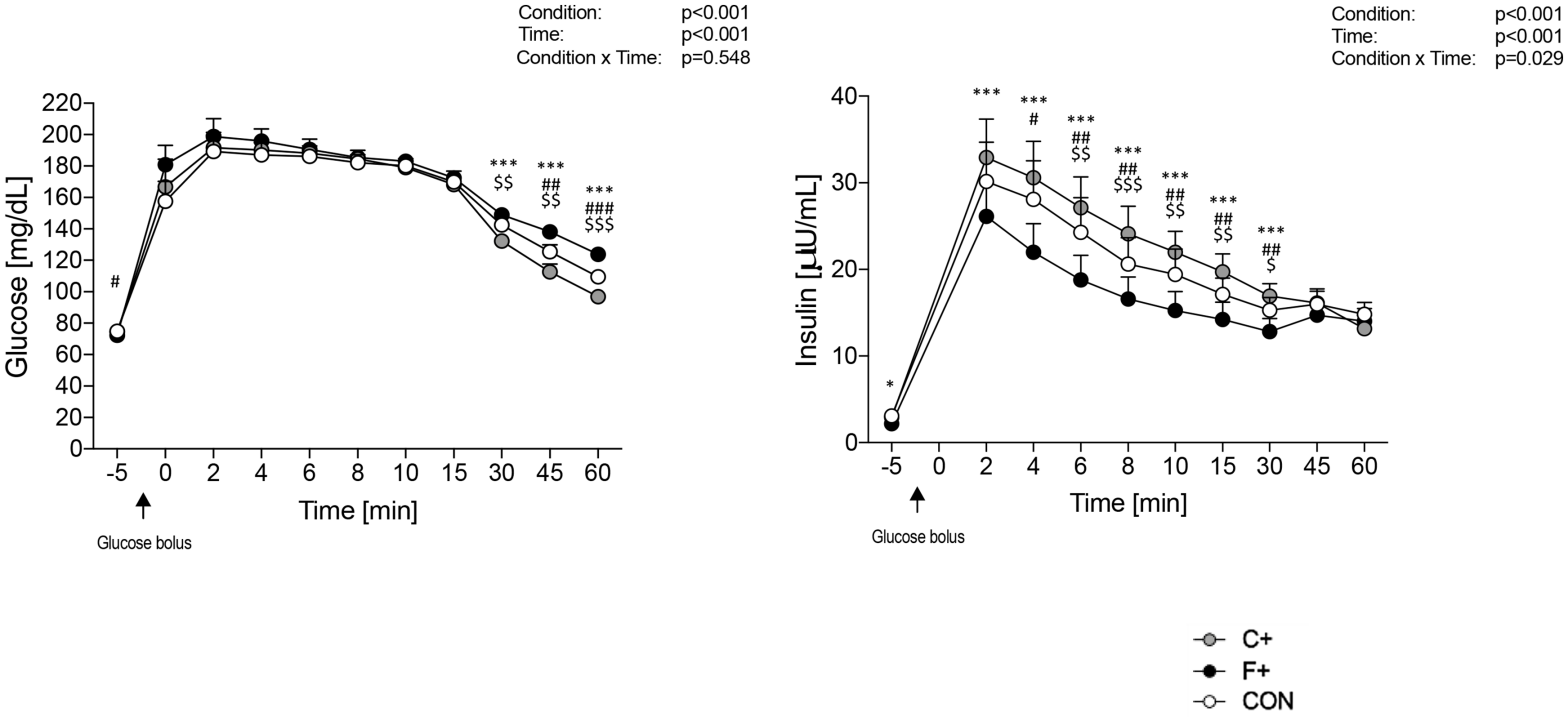
Glucose profile (A) and insulin profile (B) during IVGTT for the three diets CON, F+ and C+. Data as mean ± SEM. Two-way ANOVA for repeated measurements and dependent *t*-test for paired samples. *F+ vs. C+, #CON vs. F+, $CON vs. C+. *, #, $ p ≤ 0.05; **, ##, $$ p ≤ 0.01; ***, ###, $$$ p ≤ 0.001. Black arrow: glucose bolus.

Figure 5 depicts the glucose and insulin profile during the ivGTT on the morning of day 3. Glucose levels increased after bolus glucose injection, with the maximum value measured at minute 2, followed by a decrease in blood glucose levels in all diets. From minute 30 onwards, there are significant differences between the three diets (*p* = 0.548 for ANOVA time × condition interaction; Figure 5A). The highest insulin levels are also measured at minute 2 but then decrease in the three diets (*p* < 0.029 for ANOVA time × condition interaction) (Figure 5B), with C+ showing the highest and F+ lowest levels.

**Figure 6:**
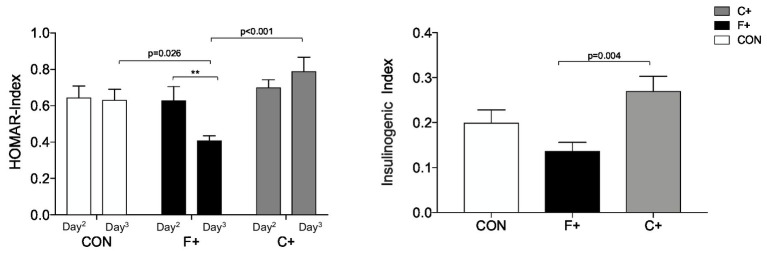
Measures of insulin resistance. (A) Plot of HOMAR index, F+ diet shows significant differences by paired *t*-test *p* < 0.01. ANOVA for the three diets showed for Day2 *p* = 0.765, Day3 *p* = <0.001; (B) plot of Insulinogenic Index by ANOVA between diets *p* = 0.005, significant differences with Bonferroni *post hoc* test. ** *p* < 0.01.

HOMA-IR and the insulinogenic index are shown in Figures 6A,B, respectively. Under F+, HOMA-IR was lower on the morning of day 3 than on day 2, being lower compared to both C+ (p ≤ 0.001) and CON (*p* = 0.026). No difference was seen between C+ and CON diets (both *p* > 0.05). The insulinogenic index differed between conditions (*p* = 0.005), with F+ showing lower values than C+ (*p* = 0.004).

## 4 Discussion

Numerous studies demonstrate a relationship between macronutrient composition and metabolic health and its long-term consequences. However, it is unclear whether even an intervention over 24 h under isocaloric diets leads to changes in glucose metabolism. To our knowledge, this study is the first human experimental study to examine short-term dietary modification in terms of an isocaloric, high-fat versus high-carbohydrate intervention diet in healthy male participants under controlled laboratory conditions. After this short-term intervention, we found increased feelings of satiety after the C+ diet without changes in hedonic behavior. Under an ivGTT, postprandial glucose and insulin levels were lower after the F+ diet than C+ and CON diets. Interestingly, lower insulin resistance in terms of lower HOMA index was observed after the F+ diet. Interestingly, the CON diet led to higher glucose levels than the C+ diet despite a lower carbohydrate content (especially sugar). In addition, increased morning fasting glucose level was found after CON and C+ diets compared to F+ diet.

Glucose homeostasis under the F+ diet showed fewer fluctuations, with blood glucose levels averaging 75–80 mg/dL. Much higher values were obtained under the CON (55% carbohydrate) and C+ (79% carbohydrate), which was predictable due to carbohydrate metabolism (39). However, the fact that CON yields higher glucose values at breakfast and lunch than the C+ diet, being significantly lower than the C+ diet only at dinner, was not expected. A tendency for F+ to have lower fasting blood glucose values than CON and C+ was observed on day 3. Brøns et al. (40) found elevated fasting blood glucose due to increased hepatic glucose production after a five-day, high-fat, hypercaloric diet. The discrepancy with our findings can be explained by the fact that a more extended intervention period and a hypercaloric diet were chosen (40). It is possible that postprandial glucose secretion changes after a more sustained high-fat diet (40).

Analogous to the blood glucose level, insulin level was subject to daily fluctuations. The rise of postprandial insulin was most evident under CON and C+, which was expected by the increased carbohydrate content of both diets. The present study did not observe an increased fasting insulin level under a 5-day high-fat diet, as described by Brøns et al. (40), which is considered a compensatory mechanism of increased hepatic insulin resistance. On the other hand, cortisol mobilizes energy reserves during physical and psychological stress and during food deprivation. This leads to elevated glucose levels to supply glucose-dependent tissues such as skeletal muscle and the brain (41). In the present study, cortisol levels showed no differences between experimental conditions, so that a stress response in the context of the intervention diet cannot explain the putative altered glucose homeostasis.

The results of the Prospective Urban Rural Epidemiology (PURE) study (42) in 2017 received much media attention worldwide, especially among consumers and patients. It concluded that a high carbohydrate intake is associated with a higher risk of total mortality, while all-cause mortality is lower for fat and individual types of fat and global dietary guidelines should be reconsidered (42). The nutritional recommendations of the expert Societies with 30–35% fat, 50–55% carbohydrates, and 15% protein intake of the total daily energy primarily consider quantitative aspects and neglect the quality of the food, i.e., the content and density of micronutrients, which are of outstanding importance for human health (43). There should be a focus on the generally excessive energy intake and inadequate diet quality, e.g., insufficient consumption of fiber-rich foods, excessive consumption of added sugar, and refined starch (44, 45). The fact that the CON diet was proposed as a “balanced” or at least recommended diet shows such a high glucose and insulin response (Figure 4).

In the ivGTT, no differences were found regarding the first-phase insulin response (FPIR) between diets. Brøns et al. (40) also found no differences in glucose levels within 30 min after glucose administration in a high-fat diet. Glucose levels in normal individuals usually peaked between 3 and 6 min at FPIR. As in Yuan et al. (46), the peak could have occurred before 3 min. Individuals with a later time to peak glucose may have a lower risk of developing T2D in the future (46). As expected, insulin levels behave analogously to glucose concentrations increasing after intravenous glucose loading. Insulin levels are significantly higher in C+ than in F+ from minute 2 to 30 during the ivGTT period. Data of CON were in between data of both other diets. This suggests an isocaloric, high-fat diet decreases FPIR and compromises insulin secretion.

In the present study, insulin resistance given by the HOMA-IR decreased on day 3 under the F+ diet compared with day 2. In contrast, HOMA-IR remains unchanged after CON, whereas it increased on C+, while both diets showed significantly higher HOMA-IR on day 3 compared to F+. Thus, one day of a high-fat diet provokes improvements in insulin resistance, even in healthy participants. Parry et al. (47) demonstrated that under a hypercaloric, high-fat diet, metabolic dysfunction in decreased insulin sensitivity can occur within a day. However, the effect may be primarily driven due to the hypercaloric diet rather than the increased fat content. This speculation can be supported by data reported by Brinkworth et al. (48) who found an improved insulin resistance in overweight subjects after an isocaloric low-carbohydrate, high-fat diet for 1 year compared to an isocaloric low-carbohydrate, low-fat diet (48). Further, Hyde et al. (49) have demonstrated an improvement in metabolic status, particularly insulin resistance, in obese patients after 4 weeks of low-carbohydrate, high-fat diets compared with isocaloric, higher-carbohydrate diets.

Even a mild positive energy balance can significantly increase body fat mass in the long run (50). Therefore, it can be assumed that an even higher energy intake, for example, in the context of cravings, will result in a correspondingly higher body weight gain.

Human appetite regulation comprises central and peripheral mechanisms that interact with environmental features. These processes are called the “satiety cascade,” which are influenced by sensory, cognitive, pre- and post-absorptive processes (51). Here, subjective feelings of hunger in response to meals did not differ between experimental diets, but satiety was significantly greater, primarily in CON and C+. Since the amount of dietary fiber in the C+ diet was the highest at 23.5 g per day, this may increase satiety. This is because chewing high-fiber foods requires time and effort, which prolongs oral intake and allows time for signals that convey feelings of satiety (52). Furthermore, Rebello et al. (52) state that the duration of oral intake plays an important role in reducing energy intake and may be comparable to gastric filling signals, which have also been shown to promote feelings of satiety. Ortinau et al. (53) saw a prolonged feeling of satiety in young, healthy women after ingesting a lower-fat meal compared with a high-fat one. Energy density and meal portion size also affect subjective satiety perception (54). Although our meals served were isocaloric and portion sizes were comparable, there was a difference in subjective feelings of satiety between the diets. Participants were the least saturated in the F+ diet, while the C+ diet had the highest feeling of satiety (Figure 2). Of note, participants of the present study were constant in body mass across study time so that changes in body fat mass could not impact found results.

The present study aimed to thoroughly examine “liking” and “wanting” for food in participants during a C+ or F+ diet compared to a CON diet using a computerized behavioral task. Our results showed a similar “liking” and “wanting” to all food categories between diets. Frank et al. (55) showed that brain activity during the “wanting” task activated a more distributed brain network than during the “liking” task. This network included taste, memory, visual, reward, and frontal regions. Furthermore, an interaction effect between dopamine depletion and the “liking” and “wanting” tasks was observed in the hippocampus. However, no BMI-related effects were found (55). A recent study of 56 subjects after bariatric surgery and significant weight loss found no differences in “liking” or “wanting” (56). In the data shown here (Figure 5), when comparing “liking” and “wanting,” it is noticeable that the rating of “wanting” was higher before meals than after meals.

Contrary to our expectations, the participants showed no differences between the three diets (CON, F+, C+) when asked to rate the sub-items of “liking” and “wanting” according to savory, sweet, and low-calorie foods (Figure 6). In the study of bariatric operated subjects, no higher “liking” or “wanting” for products rich in fat or carbohydrates was found (56). The systematic review by Oustric et al. (57) showed that food rewards changed in most weight management interventions; both “liking” and “wanting” for high-energy foods decreased after the intervention. However, few studies assessed this association and showed that a decrease in preference for high-energy foods was associated with a reduction in body weight or fat mass (57). The fact that food preferences can change with sensory appetite in a 24-h diet in healthy subjects was shown by Griffioen-Roose et al. (58). After a savory diet, the intake of sweet foods was higher than savory foods, and after the sweet diet, savory foods tended to be preferred (58).

Through indirect calorimetry data, RQ can be used to estimate the organism’s primary energy source. On the one hand, this depends on the macronutrient composition of the diet (59). The RQ results here differed significantly between diets on day 3 (Table 1); as expected, RQ was lowest under the F+ diet. This can be measured as a protective factor against fat accumulation and obesity since the organism preferentially consumes fat as an energy source (60).

Several limitations need to be discussed. Our study population comprised young, healthy, normal-weight men. Therefore, our results cannot be extrapolated to other age, gender, and BMI groups. As we studied healthy participants with normal glucose metabolism, our results need further confirmation in participants with altered metabolic regulation, e.g., patients with obesity, insulin resistance, or T2D. However, we believe that the experimental design and the well-controlled protocol and meals contribute to a better understanding of the effects of short-term dietary modification on human energy homeostasis.

## 5 Conclusion

In summary, a 24-h isocaloric, high-fat diet, in particular, led to a change in glucose metabolism but did not influence hedonic eating behavior in young, healthy men. Postprandial glucose and insulin levels were lower on the F+ diet. The CON diet led to the highest glucose levels, although the carbohydrate content, especially the sugar content, was lower than on the C+ diet. Based on this, a F+ diet could be used as a possible nutritional therapeutic approach for short-term impaired glucose tolerance patients. Further clinical trials will be necessary to verify the transferability of the results to other population groups.

## Data Availability

The original contributions presented in the study are included in the article/Supplementary material, further inquiries can be directed to the corresponding author.
