# Supplementary material for: Short-term high-fat and high-carb diet effects on glucose metabolism and hedonic regulation in young healthy men
Source: Front Nutr. 2024 Oct 29;11:1469230. doi: 10.3389/fnut.2024.1469230 (PMC11554484; doi:10.3389/fnut.2024.1469230)
Supplement: Supplementary file 1 [file Image_1.pdf]

Short-term high-fat and high-carb diet effects on glucose metabolism and hedonic regulation in young healthy men

Frontiers in Nutrition

Appendix A

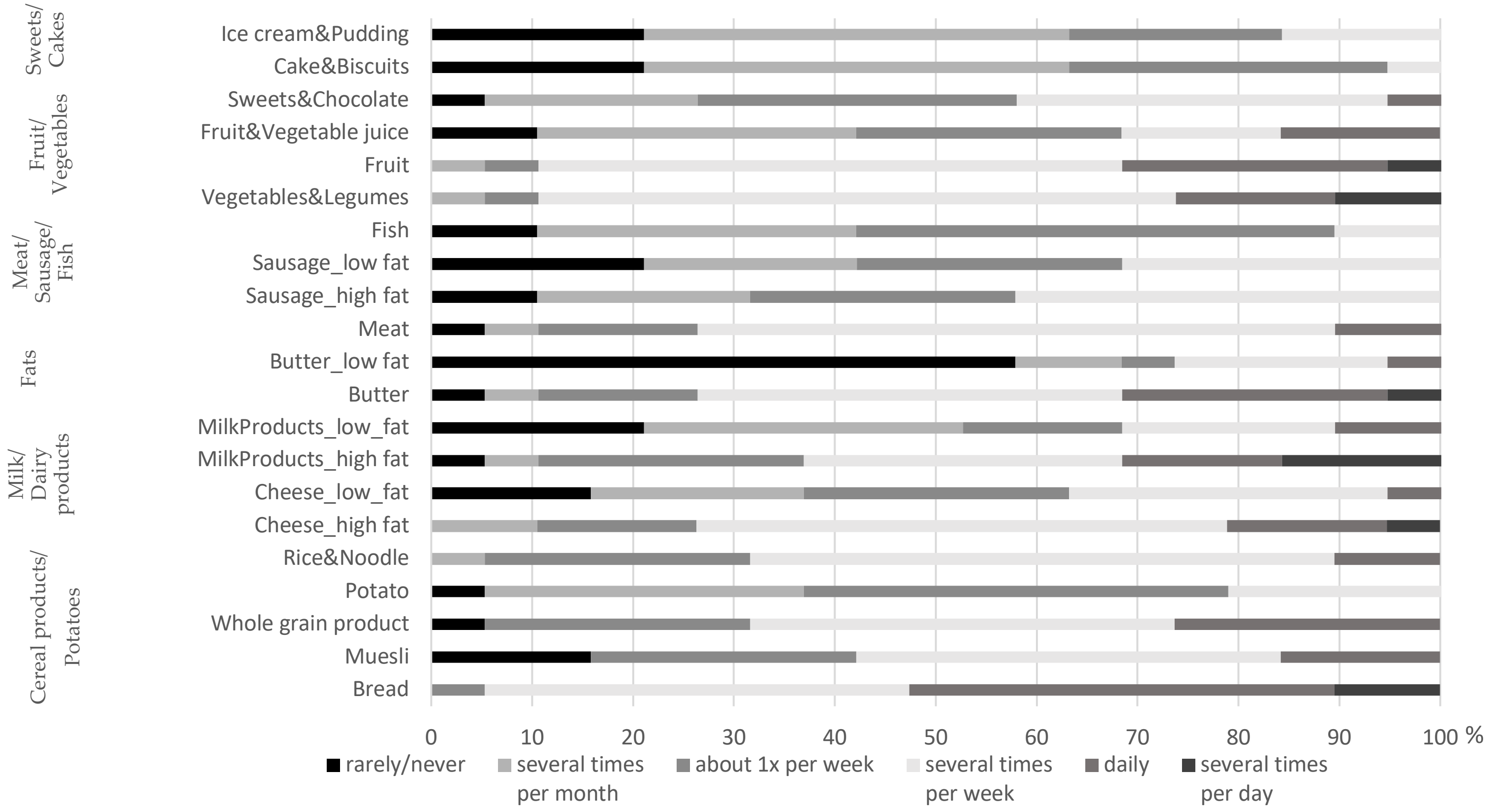

Figure A1. Dietary habits of participants, overall representation for three diets, as there are no significant differences between groups.
